# Learning in the informal spaces and re-signification of the existence of undergraduate students of nursing

**DOI:** 10.1590/0104-1169.3265.2474

**Published:** 2014

**Authors:** Isabel Silva de Jesus, Edite Lago da Silva Sena, Luana Machado Andrade

**Affiliations:** 1 Master's student, Universidade Estadual do Sudoeste da Bahia, Jequié, BA, Brazil; 2 PhD, Full Professor, Universidade Estadual do Sudoeste da Bahia, Jequié, BA, Brazil; 3 MSc, Professor, Faculdade de Tecnologia e Ciências, Guanambi, BA, Brazil

**Keywords:** Learning, Education, Nursing, Education, Higher, Philosophy, Nursing

## Abstract

**OBJECTIVE:**

to describe the perception of lecturers and undergraduate nursing students regarding the dialogic experience in the informal spaces and its relationship with training in health.

**METHOD:**

experiential descriptions were collected in the context of a public university in the non-metropolitan region of the state of Bahia, Brazil, using open interviews. These descriptions were analyzed according to the principles of the phenomenology of Maurice Merleau-Ponty.

**RESULTS:**

it was revealed that the informal spaces contribute significantly to the construction of knowledge and professional training strengthening teaching and promoting the re-signification of the subjects' experience.

**CONCLUSION:**

it is evidenced that the dialogic experience has relevancy for rethinking the teaching-learning process in the university, such that the informal spaces should be included and valued as producers of meanings for the personal and academic life of lecturers and students, with the ability to re-signify existence.

## Introduction

The study emerged from the perception that the dialogue in the informal spaces needs to be recognized in the university context as an opportunity for the (re) production of knowledges which contribute to the professional's training in health, and, especially, in nursing, both in relation to the development of personal competency in the affective domain, emotional intelligence, and intersubjective and humanitarian relationships, and in relation to technical and professional competency, as it favors the articulation of theoretical/scientific knowledge with routine experiences, with a view to the construction of skills for the planning and carrying out of effective practical actions in accordance with the emerging social demands. 

In this study, the informal space is understood as all the spaces of the university context, including the university campus and its surroundings, in which the educators and those receiving education come together without the specific aim of developing knowledges in a systematic way, for example, canteens, patio areas, benches distributed around the campus, stairways, luncheonettes, bakeries, bars near the campus, as well as spaces which are recognized as set aside for formal education, such as the library, laboratories, classrooms and others, so long as they are used for informal conversations.

Learning in the informal spaces goes beyond the limits proposed by formal, technicist and instrumentalist education, disassociated from the sociocultural context, and is presented as an innovative mode of learning, which provides an intellectual instrument which is more concrete and closer to the social practices, permeating the action and the reflection, aiming for the construction of the subjects' citizenship and critical sense^(^ 1 ^-^ 2 ^)^.

It is unacceptable that at the current time the person graduating from courses in the healthcare area should be unable to undertake humane care in a contextualized way, in the light of the complexity which engenders it, requiring competencies and skills which promote health in its integrity, which cause them to see the problems and collaborate for their solution, and which value the person as a citizen^(^ 3 ^)^.

In our academic experience, the knowledge shared in the informal spaces contributed to the teaching-learning process, principally in relation to the adopting of more critical and reflexive attitudes, to the development of communicational and relational skills, interdisciplinary experience with various areas of knowledge such as education, philosophy, and art, among others, which we can raise in the training, in the professional practice, and also in personal life. 

Based on these experiences, we have come to believe that the personal experiences which involve the routine of the academics need to be capitalised upon by students and lecturers throughout the training, so as to extend the educational settings and value the experience as an enriching and driving force of the pedagogical process in the construction of knowledge. 

To this end, we have defined as the objective of the study which was the basis for this article the description of how lecturers and students on the undergraduate nursing course perceive the dialogic experience in the informal spaces of the university context, and the relationship which they establish (if they do) with training in health. 

In his work *Phenomenology of Perception*, the philosopher Maurice Merleau-Ponty refutes the belief that there is an interior subjectivity conferring individuality on the person, distinguishing her from others. It is exactly this understanding that Merleau-Ponty tries to overcome, inserting the theory of intersubjectivity, according to which, the sensitive nature confers on the man a generality and, through this, he is always interpersonal and intersubjective^(^ 4 ^)^.

In this regard, Merleau-Ponty's theory is perfectly suited to supporting the present study, as it deals with describing perception and, for the philosopher, existence is inscribed in perceptive experience, and this is effected through dialogue, which is completely intersubjective. As a consequence, the knowledge produced in this study does not aim to explain the results achieved, but to describe the experiences which are perceived through the intersubjectivity between researchers and study subjects. 

The study's relevance lies principally in providing theoretical support, based in the experiences of lecturers and students of nursing, such that the subjects involved in the teaching-learning process in the areas of health may use the experiences of the university routine in the ambit of the informal dialogues, so as to strengthen the formal curriculum plan and produce knowledge capable of transforming the praxis of the work in health. 

## Method

This study is of a qualitative and phenomenological nature. A total of four lecturers and five students from the undergraduate course in nursing at a public university in the non-metropolitan region of Bahia participated in the study. All were female. The process of obtaining the experiential descriptions took place on the university campus during single meetings, at different times for lecturers and students, with a view to grouping the experiences of people who participate in the same social group. 

The students were selected in the following way: presentation of the research project to the Nursing groups from the sixth semester onwards, considering that from this point onwards they already have more experience of participation and coexistence in the informal spaces in the university context and, through this, are better able to describe the dialogic experience related to academic training; invitation to participate in the study; and the random selection of 10 students among those who accepted the invitation, considering the inclusion of the sixth to ninth semesters of the undergraduate course. The groups were informed that only the first five people chosen would participate in the research, so as to facilitate the group interview, ensuring the participation of all; the others would be invited if necessary.

For the selection of the lecturers, we used the following criteria: to be teaching professionalizing courses from the sixth semester onwards. Following that, we undertook a random selection of 10 lecturers, and the first five considered were invited to participate in the study. When the first selected could not participate, we invited the subsequent ones. Although we arranged the meeting with five lecturers, on the day of the interview only four attended.

In this process, the interview was used. We held meetings separately with each category, lecturers and students. The interview was supported by a script with the following guiding themes: What does dialogue in the informal spaces of the university and its surroundings mean for you? Do you think that the informal conversations in these spaces contribute to the academic training? If you believe that these conversations in the informal spaces contribute to the academic training, are they recognized as such and valued by the lecturers and students? 

The group interview occurred in a processual and intersubjective way; as the questions were put by the researcher, the participants responded in the dialogic way, without necessarily obeying an order, considering that, in the intersubjectivity, the account of one subject mobilizes that of the other. The group was mediated by one of the authors, who intervened so as to deepen and clarify questions, with a view to achieving the objective proposed. 

In all the stages of the research, the ethical precepts were respected, the study being approved by the Research Ethics Committee of Southwest Bahia State University under protocol N. 008/2011. The data were collected following consent, in writing, by the subjects. In order to maintain confidentiality in relation to the subjects' identities, they chose codenames to represent themselves, with the lecturers choosing the names of flowers and the students, the names of cartoon characters.

The interviews were recorded and transcribed in full, and the description of the perceptive experience was performed through ambiguity analysis, a technique developed for the analysis of empirical descriptions that have as their theoretical and philosophical matrix the Maurice Merleau-Ponty's philosophy of experience. This technique occurs under the perspective that, while we are reading the experiential descriptions, we make an effort to convert what has not been reflected upon to reflection and articulate a thinking to be aimed for, thrown to the exterior as a perceived object^(^ 5 ^)^.

Invested in these philosophical assumptions, based in phenomenology, it is said that the researcher is in a regime of phenomenological reduction, given that she is convinced that she is facing dogmatizing theses, characterized by convictions that the things and the others are already in themselves, that is, that they are *a priori* objectivities. However, in undertaking the exhaustive reading of the material, the researcher becomes convinced that, in spite of the ambiguities, this being a matter of perceptive experience, which is inserted in a phenomenal field, objectifications are effected as significant operations. These consist of a transmutation of the pre-reflexive pole to the reflexive, a process undertaken through speech, using words, forms, synthesis and a literary genre, to which one can add the style of the writer and the feelings inside her^(^ 5 ^)^. 

## Results and Discussion

The human being is in a constant search to confirm meanings on life and re-signify existence, both personal and collective, with the main tool used for achieving this objective being the construction of knowledge. In this regard, the dialogic experience is essential in the relationship with the similar and in the creation of contexts of intersubjectivity - characteristic aspects of the informal spaces - as when people come together without a defined purpose, the dialogue flows spontaneously, and thoughts articulated in the conversations in the routine experiences are converted into existential meanings. 

The account below evidences the wish that the educator should become closer to the scientific knowledge of the routine experience, given that the knowledge, the closer it is to the practical knowledge and to the experience, becomes more logical and meaningful. 

*[...] What is most important in the training in health, in our training as nurses, is to contextualize what we learned. [...] There is no better place to contextualize matters than in our daily experience, in our conversations, when we report our experiences. When we build knowledge in the informal space, we relate it more to our context, for example, I was talking about social security in the bar. If it were an issue which arose here in the classroom, we would begin with legislation, the rights... There, it began with me talking about my family, about how we have to find jobs soon. My mother and father do not pay into the social security system... So that is how the subject came up, that is to say, this construction of knowledge becomes more personal, related more to our context, and is closer to our experience* (Elmyra)

The description reveals the students' wish that the educator should bring the scientific knowledge closer to the routine experience, given that the closer the knowledge is to the practical knowledge and to the experience, the more logical and meaningful it becomes. In addition, the valorization of the empirical experience promotes development that is not only academic but which is also personal, which expands the creative potential and intensifies the participation of the person being educated in the teaching-learning process, when they perceive that the experience of life is not disassociated from the training, but that it has a significant contribution^(^ 6 ^)^.

As a result, Freirean thinking emphasizes that the role of the educator is not to lecture regarding his vision of the world or to try to impose it on the person being educated, but to dialogue with her regarding the two points of view, constituted by the action of the respective actors in the world^(^ 1 ^)^, as everything which one knows regarding the world, even through science, is based on a point of view or on experience, without which the scientific symbols mean nothing^(^ 7 ^)^.

We must not, therefore, limit learning only to the act of assimilating content, but serve its basic purpose, which is the comprehensive development of the human being, a phenomenon which occurs not only in the relationship between the person educating and the person being educated, but in the various contexts of intersubjectivity which are established, both in monological reflections, such as those resulting from contact with other people and, in this way, the person learns and re-learns in the relationship. 

Similarly, one finds in the accounts the importance of not only valuing life experience but also of valuing the routine of the health services as a way of contributing to the meaningful learning. On the other hand, the excessive theorization of the content and the lack of contextualization with the reality is criticized.

*It is pure theory and theory does not build as much as we do with practice, with our lives, in our context, with things which are more real, closer, it is this that helps us to make this connection, more than theory, which is put, which was placed, which was studied and is brought to our life. So I think that it is one meaning which it brings. It brings life to its theory.* (Bubbles)

*The learning stops being pleasurable because we arrive at work and see that everything is so different from what we learn. Theory is one thing and in the reality, we end up forgetting the theory and, as the practice periods are so fast and few, we have to take advantage of that time to learn what the profession obliges us to, and that's it. There is only time for this, and we forget. Let's forget it and let's go! There are things which we don't see in the classroom because it is theory, sometimes there are discussions, but the informal spaces promote the conditions that the classroom does not give and it is there that people let their hair down and speak, they identify with that environment somehow and feel more at ease to give opinions, criticize, we learn to have a critical sense.* (Little Prince)

When the students are placed in contact with the professional practice and the reality of the services, starting with their entrance to the college, they expand the environment of learning to beyond the classroom, using the context as a theoretical-conceptual basis, which develops a more concrete view of the complexity of the health system, management and organization of the services, and broadens the individual approach for the understanding of the needs of the population groups^(^ 8 ^)^.

The spaces in which the courses propose to train nurses need to be filled with scientific knowledge articulated with the routine of the work, so as to surprise the students, mobilize their imagination and produce science without falling into objectivism and banalization of the scientific and technological content. With this understanding, it is possible to value the culture, popular knowledge, experience and freedom of discussion, which favors the recovery of the pleasure of learning, as well as the social responsibility for creation, sharing and publicizing of science with a view to conscientious, citizenship-based action^(^ 9 ^)^.

The lecturers who participated in the study emphasize the positive effects of student participation in spaces besides the classroom in the academic training, for example, in research projects and expansion projects, which aim for the full development of the person being educated and the preparation for exercising citizenship, in addition to qualification for work. 

*He [a student with experience in research projects] is always seeking to humanize the environment due to the experience which he has with this type of patient [psychiatric]. He shows details that the nurse who was there did not perceive, and he, after one week on the placement, was already carrying out situational diagnosis and identifying what is really serious.* (Carnation)

*I have the example of one student, who I am supervising in her undergraduate end-of-course paper, [who] was a grant-funded researcher in my team and who is continuing with the same study object in her Master's degree. Actually, there was a sequence, and we notice that there has been an increase, that there is this involvement that strengthens this professional training, enriches it, and gives a better basis.* (Rose)

In this perspective, the participation of those being educated in non-obligatory activities encourages the seeking of information and the process of self-learning, involving various situations such as the identification, analysis and resolution of problems, in different scenarios, both in the academic space and in the health services and community; it also encourages critical and reflexive discussion regarding the practices undertaken, with a view to transforming these^(^ 10 ^)^.

The non-obligatory activities, which include actions of research and extension, are also commonly shared among the academics in the informal spaces, which allows students who are not engaged in these projects to extend their knowledge on various issues, as well as being awoken to and motivated for integrating into such activities as well. 

*In the first terms, a class finished and I would go home straightaway because I thought that I would lose time if I stayed talking here at college, that I would not be studying. It is just that, on the contrary, I didn't know anybody, only my class-mates. Today I know more people and I see that I was wasting my time by being so restricted to studies, because I think that dialogue allows you to know about a course which you are going to have, an event, or a project which you can participate in. So, on the contrary, in addition to you thinking that you are going to waste time, no, you are going to be learning about new things, something which has changed...* (Tinkerbell)

Through involving themselves in these activities, the students have the opportunity to develop new reflections which the curricular plan cannot promote, such as to develop personal projects and actions which are different from the routine ones, and come to be engaged in something which is closer to their existential interests, as well as to experience innovative experiences which can strengthen the formal curriculum and provide creative elements for the formal academic activities^(^ 3 ^,^ 11 ^)^.

The accounts revealed that the academic trajectory that includes the informal experiences in the university environment has fundamental importance in the construction of the human being, as it extends beyond the technical training, produces meaning, and re-signifies the existence of students and lecturers. 

*I usually say that I took advantage of everything that University had to offer me. For me, this is a space for personal, political and collective growth, a vision of the collective, of work, of professional relationships. Because one student mentor position which we get, is a grant we get, which faces challenges of working with the other... All of this is growth.* (Elmyra)

*We don't stop to think that this [the dialogue in the informal spaces] is so important, and that it will contribute so much to training a professional, that maybe it would be an essential factor. If I had not had this during my training, if I had not participated in the spaces, I would not be the professional I am going to be in the future, because a lot of the experience, a lot of that which I can conciliate in my professional practice, I learned there, during those conversations, during an experience.* (Bubbles)

*I had the opportunity to experience situations which were striking in my life, and they made me reflect on my way of being as a human being. It was one of the most gratifying experiences which I had in the eight years of university as a lecturer. After I experienced this, for sure, my way of being and of facing things, not only the students, but life too, became different. I am grateful for having had the opportunity, and grateful because it made me grow as a professional and as a person. And this was not formal knowledge, it really came from informal conversations, where this dialogue was established, it is a dialogue of reliability, of confidences even, where there was respect for him [student] and for me, as a human being.* (Lily)

The academic context offers a universe of options for the student such that he may fully develop, although not all take advantage of this. Although the educational experience in university challenges and produces growth in a processual and imperceptible way, this process does not occur in a linear or vertical way which keeps us up to date, but occurs in a tangle of possibilities and unanticipated transmutations resulting from creativity^(^ 12 ^)^. 

The opportunities for learning and growth affect not only the students but also the lecturers, when these establish relationships with the students in the academic routine, a phenomena that Merleau-Ponty defined as *I can*, the experience of becoming the *other* without losing one's identity as a person^(^ 4 ^)^. It is a process of transcendence, which occurs through the intersubjectivity that is characteristic of the dialogic relationship. The subjects involved establish a relationship of complicity, share feelings, reveal their humanity and become an intercorporal generality. Lecturer and students can come together and reveal an identity in the difference.

One lecturer emphasizes the limitation of the formal space of the classroom for promoting intersubjectivity, but understands that time produces maturing so as to perceive that the experiences of life allow learning, and values the informal environment as an opportunity for knowing the other. In addition, it was also emphasized that coexistence strengthens the social relationships and allows the dissolution of prejudices. 

*Time and experience cause us to mature, and we learn with each student, each experience, with each group with which we work. So this leads us to reflect and to see how important these spaces are, because we know the other, while this doesn't happen in the class-room.* (Sunflower)

*When the placement began, the student was already labeled, this one will be good, this one will be bad! Later I began to perceive that I had to begin to change this from the first day, that I could not work in this perspective, because by the end of the placement I always managed to spot the strengths, the abilities, and even began to notice the professional insertion of some of them who I had thought didn't have all this ability. So I began to get closer to them, to talk to them more, really to seek this trust, this friendship. Today I don't do this anymore, my way of being has changed a lot. In the last few years I have really managed to have this new perspective.* (Rose)

Becoming closer to, and gaining information about, the person with whom one coexists is essential for establishing social relationships of trust, even those which are more formal, such as those at work, as lack of knowledge can create false expectations in relation to the other's capacity, leading one either to undervalue or overvalue them - the natural tendency of the human being to make a value judgment, whether positive or negative. 

In this regard, the result of the lecturers' experience in the context of life was positive, as it allowed them to change their worldview, converting prejudice into creative strength, and that experience with the other revealed their perceptions and did not allow that predetermined concepts should guide their work, way of being, and their relationships with the students. 

The experience, at the same time as it revealed the need for creating strategies to mobilize the strength which exists in each student as a human being, made the reconstruction of the personal and professional identity of the educator essential. The event was able, according to Merleau-Pontyan thought, to dissolve opacities and request to the body the attention of the whole existence, also confirmed by the discrepancies which caused them to see the facticity of the man and of the world^(^ 7 ^)^.

In this perspective, the lecturers also considered that the informal spaces favor communication and help the students to cope with challenges and routine changes, in addition to being an agreeable option for production of knowledge, and promoting health and well-being.

*We can go anywhere on earth and understand, as a process of adaptation, something which we learnt in university, we will be able to put into practice the knowledge which we absorbed here, what is to come is construction, challenge, a learning! These are things which we will have all our lives* (Little Prince)

*For me, the construction of the dialogic experience in the university context and the training of the health professional in the informal spaces is this: well-being! It is health, it is construction of knowledge as a positive thing, pleasant, agreeable. Actually, first choice, because often, in the construction of knowledge... For example, for us to choose to come to our college is a question of need, to specialize in order to do well professionally. But I understand this construction in the informal spaces as an agreeable thing, as a thing which we choose, not through need, but for well-being.* (Elmyra)

It is through taking the experiences and practices of the subjects involved as a starting point that informal learning has gained territory in pedagogical discussions, and the repercussions are being gradually extended, contributing to the construction of bridges between popular knowledge and scientific knowledge in the university experience, which characterizes a process of training beyond technical learning, but a training for life, in which the graduates feel more secure for facing future challenges^(^ 3 ^,^ 6 ^)^.

As a result, the informal spaces, in the context of health training, can constitute a resource of great potential for a broader training, producing actions and interactions which dimension the link between the theoretical and the experiential, bringing the world of work closer to the world of life. 

The broader training must bring together other modes of knowledges in the domains of ethics, esthetics, affectivity, hospitality and co-existence. This being the case, the intercession of formal teaching - didactic contract of content and behaviors - with the informal - the subjective and structural characteristics of the environment of classes and surroundings that contribute to or influence the process of education socialization, making clear the coexistence of collateral learning with the formally explicit curriculum^(^ 13 ^)^ - will permit the comprehensive and more humanist training, as it favors the construction of the professional and existential identity^(^ 14 ^)^.

In the light of this, the students' experiential descriptions caused us to believe that the theoretical and practical knowledge necessary for the nurse's training extend beyond the formal curriculums of undergraduate courses, postgraduate courses, continuous education at work and others which provide knowledge and technical skills to deal with the varying situations in the health field. As a result, the construction of the career must interlace with the subjects' growth, as people who are more open to learning in life tend to develop meaningful works, allying professional realization with personal satisfaction.

## Conclusion

The results reveal that the informal spaces are perceived as promoting significant knowledge which strengthens the formal teaching and favors the re-signification of the experience of the subjects throughout their lives, learning and work. As a result, they contribute to the training of the nurse, but also to the training of the other health professionals, to the extent that they produce intersubjectivity and bring together knowledges and virtues, providing a broader professional construction, as the learning is interlaced with the subjects' life experiences. 

However, for them to be used fruitfully, it is necessary for academia to recognize the potential of the informal spaces, and encourage the students to establish dialogic relationships in contexts of intersubjectivity during their training. This being the case, the study is shown to be relevant for rethinking the teaching-learning process in the university, such that the informal spaces may be included and valued as producers of meanings for the personal and academic life of students and lecturers, with the capacity to re-signify existence.

The phenomenological perspective for the informal spaces in the context of the training of the nurse showed that the production of knowledge and learning in these spaces goes beyond that established in the undergraduate, postgraduate, and educational courses run in the workplace - whose focus is the technical-scientific aspect - as they bring together other competences and essential virtues for care of oneself and for the other, as in the example of generosity, respect and tolerance, which lead to the re-signification of existence.
